# Water Extract of *Capsella bursa-pastoris* Mitigates Doxorubicin-Induced Cardiotoxicity by Upregulating Antioxidant Enzymes

**DOI:** 10.3390/ijms242115912

**Published:** 2023-11-02

**Authors:** Yuhui Jeong, Sun-Ho Lee, Jangho Lee, Min-Sun Kim, Yu-Geon Lee, Jin-Taek Hwang, Sang-Yoon Choi, Ho-Geun Yoon, Tae-Gyu Lim, Seung-Hyun Lee, Hyo-Kyoung Choi

**Affiliations:** 1 Korea Food Research Institute, Wanju-gun 55365, Republic of Korea; j.yuhui1@kfri.re.kr (Y.J.); jhlee@kfri.re.kr (J.L.); mskim@kfri.rekr (M.-S.K.); ugun2@kfri.re.kr (Y.-G.L.); jthwang@kfri.re.kr (J.-T.H.); sychoi@kfri.re.kr (S.-Y.C.); 2 Department of Food Science & Biotechnology, Sejong University, Seoul 05006, Republic of Korea; tglim@sejong.ac.kr; 3 Department of Biochemistry and Molecular Biology, Graduate School of Medical Science, Brain Korea 21 Project, Yonsei University College of Medicine, Seoul 03722, Republic of Korea; shlee0626@yuhs.ac (S.-H.L.); yhgeun@yuhs.ac (H.-G.Y.); 4 Institute of Genetic Science, Yonsei University College of Medicine, Seoul 03722, Republic of Korea; 5 Division of Cardiology, Department of Medicine, Johns Hopkins University, Baltimore, MD 21205, USA

**Keywords:** doxorubicin-induced cardiotoxicity, human-induced pluripotent stem-cell-derived cardiomyocytes, superoxide dismutase, mitochondrial dysfunction, *Capsella bursa-pastoris*

## Abstract

Doxorubicin (DOX), an effective chemotherapeutic drug, causes cardiotoxicity in a cumulative and dose-dependent manner. The aim of this study is to investigate the effects of hot-water extract of *Capsella bursa-pastoris* (CBW) on DOX-induced cardiotoxicity (DICT). We utilized H9c2 rat cardiomyocytes and MDA-MB-231 human breast cancer cells to evaluate the effects of CBW on DOX-induced cell death. Superoxide dismutase (SOD) levels, reactive oxygen species (ROS) production, and oxygen consumption rate were measured in H9c2 cells. C57BL/6 mice were treated with DOX and CBW to assess their impact on various cardiac parameters. Human-induced pluripotent stem-cell-derived cardiomyocytes were also used to investigate DOX-induced electrophysiological changes and the potential ameliorative effects of CBW. UPLC-TQ/MS analysis identified seven flavonoids in CBW, with luteolin-7-O-glucoside and isoorientin as the major compounds. CBW inhibited DOX-induced death of H9c2 rat cardiomyocytes but did not affect DOX-induced death of MDA-MB-231 human breast cancer cells. CBW increased SOD levels in a dose-dependent manner, reducing ROS production and increasing the oxygen consumption rate in H9c2 cells. The heart rate, RR interval, QT, and ST prolongation remarkably recovered in C57BL/6 mice treated with the combination of DOX and CBW compared to those in mice treated with DOX alone. Administration of CBW with DOX effectively alleviated collagen accumulation, cell death in mouse heart tissues, and reduced the levels of creatinine kinase (CK) and lactate dehydrogenase (LDH) in serum. Furthermore, DOX-induced pathological electrophysiological features in human-induced pluripotent stem-cell-derived cardiomyocytes were ameliorated by CBW. CBW may prevent DICT by stabilizing SOD and scavenging ROS. The presence of flavonoids, particularly luteolin-7-O-glucoside and isoorientin, in CBW may contribute to its protective effects. These results suggest the potential of CBW as a traditional therapeutic option to mitigate DOX-induced cardiotoxicity.

## 1. Introduction

Doxorubicin (DOX), an effective anthracycline drug, is generally prescribed as a chemotherapeutic agent for treating various human cancers such as breast cancer, solid tumors, lymphoma, and leukemia [1]. However, its clinical applications are limited because of its cumulative cytotoxicity [2]. DOX induces significant cardiotoxicity, consequently triggering acute and chronic cardiac disorders, such as congestive heart failure, cardiomyopathy, and myocardial injury, in patients receiving cumulative doses [3,4].

DOX-induced cardiotoxicity (DICT) is multifactorial and occurs through oxidative stress, mitochondrial dysfunction, autophagy, DNA damage, lipid peroxidation, and calcium overload [5,6,7]. Although the exact mechanism of DICT has not been completely elucidated, oxidative stress resulting from excessive generation of reactive oxygen species (ROS) is considered a major driver [8]. The main contributor to ROS generation following DOX exposure is the redox cycling of DOX-derived quinone-semiquinone in complex I of the mitochondrial electron transport chain [9]. DOX exhibits high affinity for cardiolipin, which forms a complex with cytochrome C within the inner mitochondrial membrane [10]. The DOX–cardiolipin complex disrupts the functionality of cardiolipin, thereby releasing cytochrome C into the cytosol and consequently initiating apoptotic cell death [11].

Antioxidant enzymes play significant roles in scavenging ROS during DICT [12]. Superoxide dismutases (SODs) consisting of three isoforms, including Cu/Zn SOD (SOD1), Mn SOD (SOD2), and extracellular Cu/Zn SOD (SOD3), are key antioxidant enzymes. Each isotype is located in a different subcellular fraction [13]. SOD2 localized in the mitochondrial matrix alleviates DOX-induced cardiomyopathy by suppressing free radicals [14]. The transcription of genes, such as *Sod1*, *Sod2*, and *heme oxygenase 1* (*HO-1*), encoding antioxidant enzymes to maintain cellular redox homeostasis is activated by nuclear factor erythroid 2-related factor (NRF) 2, a basic region leucine zipper transcription factor, thereby preventing ROS accumulation [15]. Under oxidative stress conditions, NRF2 dissociates from its complex with Kelch-like ECH-associated protein 1 and translocates to the nucleus, where it binds to the antioxidant response elements of genes, consequently upregulating the expression of antioxidant enzymes [16]. DOX decreases the expression of NRF1, NRF2, and HO-1 [17,18].

*Capsella bursa-pastoris* (L.), commonly known as Shepherd’s purse, is a well-known traditional herbal medicine used to treat conditions like edema, conjunctivitis, and metrorrhagia [19]. This plant has pharmacological effects, such as lowering blood pressure, improving eyesight, and promoting hemostasis [20]. Traditionally, this herb has been employed in medicine as a hemostatic, hypotensive, diuretic, astringent, stimulant, and anti-ulcerogenic agent [21]. Additionally, this plant has been used in the treatment of cataracts and hemorrhoids in China and Anatolia for many centuries [22,23]. *Capsella bursa-pastoris* (L.) contains various chemicals including flavonoids, alkaloids, sterols, fatty acids, and amino acids [24]. The variety in its composition is considered to provide a broad spectrum of beneficial effects on human health, including anti-inflammation, anticancer, and antioxidant properties and efficacy against metabolic diseases [25]. However, whether *Capsella bursa-pastoris* (L.) exerts a cardioprotective effect against DOX remains unknown.

To investigate this, we observed various parameters, such as electrocardiography and serum and histopathological analyses using an in vivo DICT model of mice. Furthermore, based on the multielectrode array (MEA) system, electrophysiological changes were monitored in ex vivo human-induced pluripotent stem-cell-derived cardiomyocytes (hiPSC-CMs). In addition, to elucidate the underlying molecular mechanism, we analyzed antioxidant enzymes and their transcription factors and subsequently verified the changes in mitochondrial function.

## 2. Results

### 2.1. Concentration of Metabolites Measured in CBW

**Figure 1:**
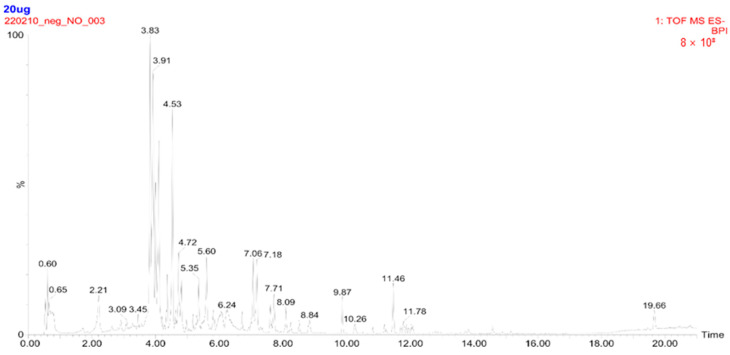
Total ion chromatogram of hot-water extract of *Capsella bursa-pastoris* (CBW) obtained by ultra-performance liquid chromatography (UPLC) triple quadrupole mass spectrometry (UPLC-TQ-MS). Twenty micrograms of CBW were injected into the UPLC system followed by TQ-MS analysis, and a total ion chromatogram was acquired over a period of 21 min. The internal standard (IS), ^13^C_3_-catechin, was used with a retention time of 3.8 min.

UPLC-TQ-MS analysis was performed to identify the major metabolites in CBW. Seven major peaks within a retention time between 4.0 and 5.5 min were identified as flavonoid glycosides, including apigenin-6,8-di-C-glucoside, isoorientin, isoquercitrin, luteolin-7-O-glucoside, narcissoside, quercetin 3-O-(6″-acetyl-glucoside), and thermopsoside (Figure 1). Concentrations of the compounds ranged from 4.26 to 133.41 μg/g (luteolin-7-O-glucoside). Among them, luteolin-7-O-glucoside (133.41 μg/g) and isoquercitrin (131.22 μg/g) were found to be rich in CBW, compared to the other compounds (Table 1).

### 2.2. CBW Protects Cardiomyocytes against DOX-Induced Cytotoxicity

**Figure 2:**
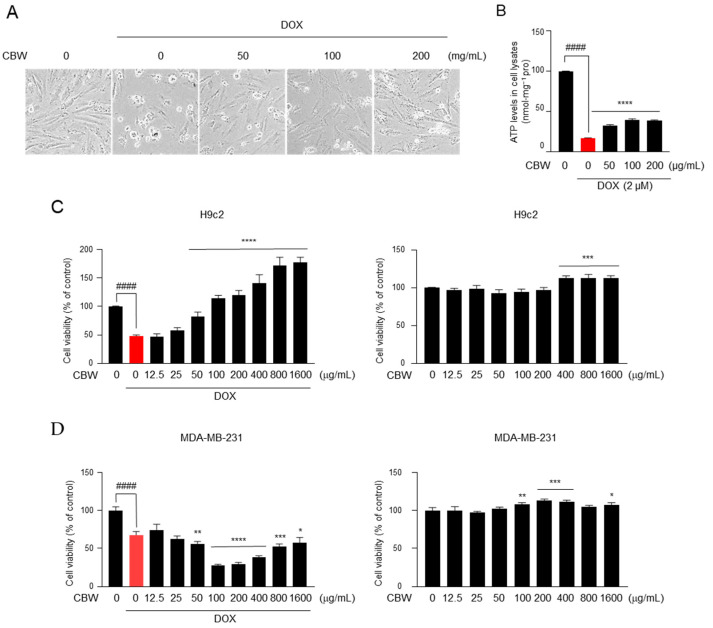
CBW inhibits doxorubicin (DOX)-induced death of rat cardiomyocytes, H9c2, without affecting the death of MDA-MB-231 breast cancer cells. (**A**) CBW protects rat cardiomyocytes against DOX-induced cell death. H9c2 cells were pretreated with CBW for 24 h. Then, 2 μM DOX was added for another 48 h, and cells were observed using an optical microscope. (**B**) CBW blocked DOX-induced ATP loss. The values are presented as mean ± standard deviation (SD) of three independent experiments. #### *p* < 0.0001 (control vs. DOX) (Student’s *t*-test); **** *p* < 0.0001 (DOX vs. CBW) (one-way analysis of variance [ANOVA]). (**C**) CBW increased the viability of H9c2 cells with (**left** panel) or without (**right** panel) DOX treatment. The values are presented as mean ± SD of three independent experiments. #### *p* < 0.0001 (control vs. DOX) (Student’s *t*-test); **** *p* < 0.0001 (DOX vs. CBW) (one-way ANOVA) (**left** panel); *** *p* < 0.001 (control vs. CBW) (one-way ANOVA) (**right** panel). (**D**) Effect of CBW on DOX-induced death of MDA-MB-231 cells. MDA-MB-231 cells were pretreated with CBW for 24 h. Then, 2 μM DOX was added for another 48 h, and cell viability was measured. The values are presented as mean ± SD of three independent experiments. #### *p* < 0.0001 (control vs. DOX) (Student’s *t*-test); * *p* < 0.05, ** *p* < 0.01, *** *p* < 0.001, and **** *p* < 0.0001 (DOX vs. CBW) (one-way ANOVA) (**left** panel). * *p* < 0.05, ** *p* < 0.01, and *** *p* < 0.001 (control vs. CBW) (one-way ANOVA) (**right** panel).

To examine whether CBW inhibits DOX-induced death of cardiomyocytes, we initially observed the morphology of H9c2 cells treated with 0, 50, 100, or 200 µg of CBW along with 2 µM DOX for 48 h. As shown in Figure 2A, cells acquired a contracted and aggregated shape after DOX treatment; however, this aberrant morphology was reduced by CBW in a concentration-dependent manner. A high ATP concentration indicates a high number of living cells. Therefore, cell viability was measured depending on ATP levels. The ATP level dramatically decreased in DOX-treated H9c2 cells, which was reversed by CBW treatment (Figure 2B). To verify these results, WST-1 assay was performed under identical conditions. Viability of H9c2 cells decreased following DOX treatment, which was reversed by CBW in a concentration-dependent manner (Figure 2C, left panel). CBW did not induce cytotoxicity in H9c2 cells (Figure 2C, right panel). Next, we evaluated whether CBW interfered with the efficacy of DOX. Interestingly, more than 50 µg/mL of CBW in combination with DOX significantly reduced the viability of MDA-MB-231 cells compared to that of the DOX treatment group (Figure 2D, left panel). CBW showed no cytotoxicity against MDA-MB-231 cells (Figure 2D, right panel). Taken together, these results indicate that CBW does not affect the anticancer efficacy of DOX and protects cardiomyocytes from DICT.

### 2.3. CBW Maintains Mitochondrial Functions by Inhibiting ROS Generation in H9c2 Cells

**Figure 3:**
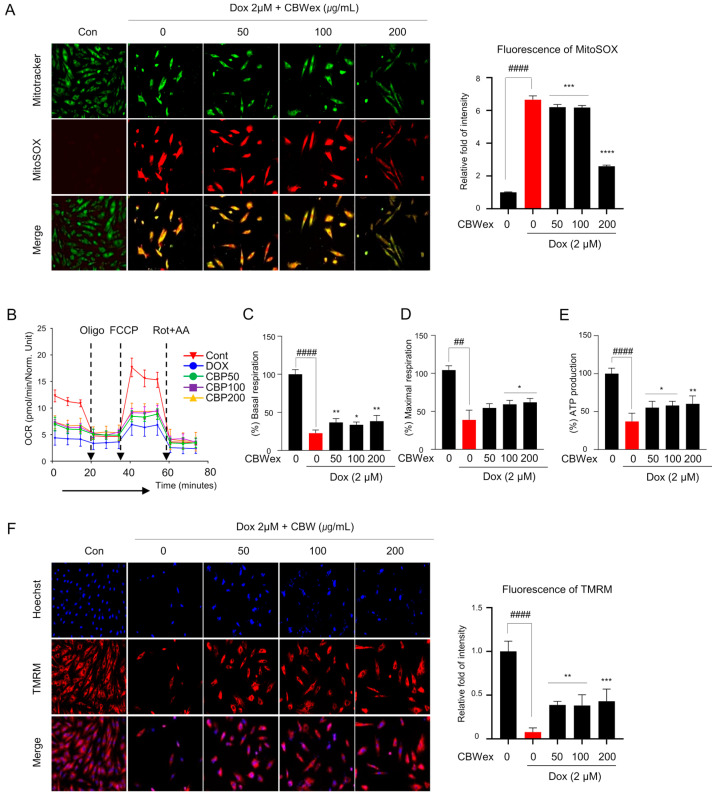
CBW reverses DOX-induced mitochondrial dysfunction. Mitochondrial reactive oxygen species (ROS) generation in H9c2 cells (200×). (**A**) Representative images demonstrating a dose-dependent decrease (**left** panel). Fluorescence intensity was quantified using ImageJ software (Ver. 1.53). The quantified intensities as the relative fold changes with respect to the control values are presented as mean ± SD of three independent experiments. #### *p* < 0.0001 (control vs. DOX) (Student’s *t*-test); *** *p* < 0.001 and **** *p* < 0.0001 (DOX vs. CBW) (one-way ANOVA). (**B**–**E**) CBW recovered DOX-induced decrease in mitochondrial respiration. Mitochondrial oxygen consumption rate (OCR) was analyzed in H9c2 cells. The arrow indicates the sequential addition of 1.5 μM oligo, 2 μM p-triflouromethoxyphenylhydrazone (FCCP), and 0.5 μM rotenone with 0.5 μM antimycin A (AA). OCR was expressed as pM O_2_/min/µg protein (**B**). Basal respiration was calculated as the difference between the pre-oligomycin and Rot + AA treatment values (**C**). Maximal respiration was converted with OCR between FCCP and Rot + AA treatment (**D**). ATP production was calculated by subtracting the pre-oligomycin treatment values from post-oligomycin treatment values (**E**). The values are presented as mean ± SD of three independent experiments. ## *p* < 0.01 and #### *p* < 0.0001 (control vs. DOX) (Student’s *t*-test); * *p* < 0.05 and ** *p* < 0.01 (DOX vs. CBW) (one-way ANOVA). (**F**) CBW reversed DOX-induced decrease of mitochondrial membrane potential in H9c2 cells (200×). The fluorescence intensity was quantified using ImageJ software. The quantified intensity was demonstrated as the relative fold change with respect to the control values, and the values are presented as mean ± SD of three independent experiments. #### *p* < 0.0001 (control vs. DOX) (Student’s *t*-test); ** *p* < 0.01 and *** *p* < 0.001 (DOX vs. CBW) (one-way ANOVA).

To test whether the effect of CBW on cardiomyocyte protection was related to inhibition of ROS production, we measured mitochondrial ROS (mROS) levels following DOX treatment with or without CBW in H9c2 cells. The mROS levels, as detected by MitoSOX staining, were significantly higher in the DOX-treated group than in the control group. The increased intensity of MitoSOX staining decreased in the CBW-treated groups in combination with DOX compared to the that in the group treated with DOX alone (Figure 3A). To ascertain whether CBW recovered DOX-induced defects in mitochondrial bioenergetics, we examined mitochondrial function. Basal respiration, maximal respiration, and ATP in real-time oxygen consumption rates were lower in DOX-treated H9c2 cells than those in the control cells, indicating low mitochondrial respiratory function. Following CBW treatment, the indicators of mitochondrial function remained relatively higher than those in the DOX treatment group but lower than those in the control group (Figure 3B). Decreased mitochondrial membrane potential following DOX treatment was restored by CBW treatment (Figure 3F). Taken together, CBW functionally reversed DOX-induced mitochondrial dysfunction by inhibiting ROS production in H9C2 cells.

### 2.4. CBW Inhibits the DOX-Induced Apoptosis of H9c2 Cells through Nrf2–SOD Activation

**Figure 4:**
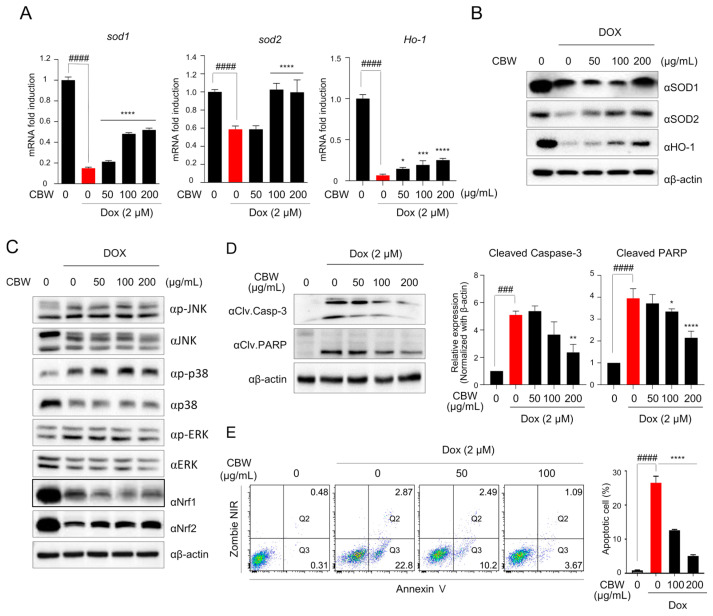
CBW prevents DOX-induced apoptotic cell death through Nrf2-mediated SOD activation in H9c2 cells. (**A**,**B**) CBW increased SOD1, SOD2, and HO-1 expression following DOX treatment in H9c2 cells. The results were demonstrated as the relative fold change with respect to the control values, and the values are presented as mean ± SD of three independent experiments. The means with different superscript letters are significantly different. #### *p* < 0.0001 (control vs. DOX) (Student’s *t*-test); * *p* < 0.05, *** *p* < 0.001, and **** *p* < 0.0001 (DOX vs. CBW) (one-way ANOVA) (**A**). Total protein was extracted from the cells and used for immunoblot assay (**B**). (**C**) CBW reversed DOX-induced Nrf2 reduction in H9c2 cells. Total protein was extracted from the cells and then used for immunoblot analysis. (**D**) CBW decreased the cleavage of caspase-3 and PARP in H9c2 cells. Total lysates were used for immunoblot analysis (**left** panel). Band intensities were quantified using Fusion analysis software (Ver. 16.07). The quantified expression was normalized with respect to that of the internal control, β-actin (**right** panel). The values are presented as mean ± SD of three independent experiments. The means with different superscript letters are significantly different. ### *p* < 0.001 and #### *p* < 0.0001 (control vs. DOX) (Student’s *t*-test); * *p* < 0.05, ** *p* < 0.01, and **** *p* < 0.0001 (DOX vs. CBW) (one-way ANOVA). (**E**) CBW blocked DOX-induced apoptosis of H9c2 cells. The rate of apoptosis was calculated as the percentage of apoptotic cells in 5 × 10^4^ cells analyzed in each of three independent experiments. The number in the corner of the Q3 or Q2 quadrant indicates the percentage of early or late apoptosis, respectively (**left** panel). The sum of the percentage of apoptotic cells is presented in bar graphs (**right** panel). The values are presented as mean ± SD of three independent experiments. The means with different superscript letters are significantly different. #### *p* < 0.0001 (control vs. DOX) (Student’s *t*-test); **** *p* < 0.0001 (DOX vs. CBW) (one-way ANOVA).

To elucidate the mechanism underlying the effect of CBW on mitochondrial dysfunction, we first observed the expression of antioxidant enzymes, such as SOD1, SOD2, and HO-1, which are involved in suppressing ROS production in the mitochondria. The mRNA levels of *Sod1*, *Sod2*, and *HO-1* decreased in DOX-treated H9c2 cells, which was reversed by CBW treatment (Figure 4A). The expression of SOD1 was decreased in the 50 and 100 µg/mL CBW treatment groups compared to the DOX-alone treatment group, and it was observed to maintain expression levels in the 200 µg/mL CBW treatment group similar to the control group. However, the expression of SOD2 increased in a concentration-dependent manner with CBW treatment (Figure 4B). Nrf1/2 is a key transcription factor that mediates the transcription of *Sod1* and *Sod2*. The Nrf signaling pathway is upregulated by the mitogen-activated protein kinase (MAPK) pathway. Therefore, Western blot analysis was performed to investigate whether CBW increased the expression of antioxidant enzymes through the MAPK–Nrf signaling pathway. As shown in Figure 4C, CBW selectively upregulated Nrf2 expression without affecting the phosphorylation of c-Jun N-terminal kinase, p38, and extracellular signal-regulated kinase (ERK). Next, to verify whether Nrf2-mediated SOD activation ameliorated apoptosis of H9c2 cells following CBW treatment in combination with DOX, the expression of cleaved caspase3 and cleaved PARP was analyzed by Western blotting and FACS analysis. DOX-induced cleavage of both proteins was reduced following CBW treatment in a concentration-dependent manner (Figure 4D). The proportion of Annexin V-stained apoptotic cells was markedly decreased by CBW treatment (Figure 4E). These results indicated that CBW protected H9c2 rat cardiomyocytes against DOX-induced apoptosis by activating the Nrf2–SOD signaling pathway.

### 2.5. CBW Protects Cardiomyocytes against DOX-Induced Cardiac Toxicity

**Figure 5:**
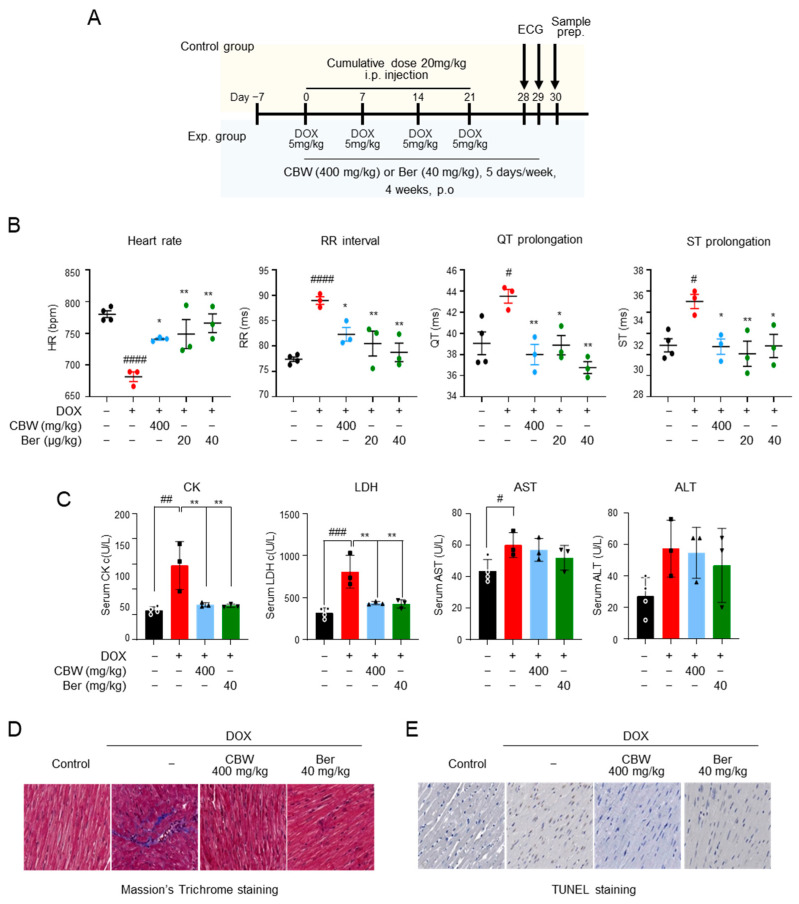
CBW protects cardiac function and diminishes cardiac injury in a DOX-induced cardiotoxicity in vivo model. (**A**) In vivo study scheme. (**B**) CBW ameliorated DOX-induced cardiotoxicity (DICT)-induced cardiac dysfunction. ECG was monitored without anesthesia using a non-invasive method. Heart rate, RR interval, QT prolongation, and ST prolongation were measured (n = 3/group). The values are presented as mean ± standard error (SE). # *p* < 0.05 and #### *p* < 0.0001 (control vs. DOX) (Student’s *t*-test); * *p* < 0.05 and ** *p* < 0.01 (DOX vs. CBW or Berberine [Ber]) (one-way ANOVA). (**C**) CBW improved DOX-induced toxicity indexes. The values are presented as mean ± SE. # *p* < 0.05, ## *p* < 0.01, and ### *p* < 0.001 (control vs. DOX) (Student’s *t*-test); ** *p* < 0.01 (DOX vs. CBW or Ber) (one-way ANOVA). (**D**,**E**) CBW administration inhibited DOX-induced cardiac injury. Cardiac fibrosis and DNA damage caused by DICT were determined using Masson’s Trichrome staining (**D**) and TUNEL staining (**E**), respectively. (magnification, 40×).

Cardiotoxicity was induced in mice by *i.p.* injection of 20 mg/kg DOX at a cumulative dose; 400 mg/kg CBW was administrated for four weeks, and ECG was performed before sample preparation (Figure 5A). The effects of CBW on ECG parameters in mice are shown in Figure 5B. The mean heart rates of the control and berberine (Ber) (positive control) groups were 780 and 766 beats/min, respectively, and no statistical significance was observed. The RR interval and QT and ST prolongation also showed no differences between the two groups. Bradycardia (681 beats/min) was observed, and the RR interval and QT and ST prolongation abnormally increased in the DOX-treated group compared to those in the control and Ber groups. DOX-induced abnormalities in ECG improved in the CBW-treated group. The heart rate, RR interval, and QT and ST prolongation recovered to levels similar to those in the Ber-administered groups. The activities of CK and LDH, which are the indicators of cardiotoxicity in serum, were measured (Figure 5C). Their activities were highest in the DOX-treated group, whereas in the CBW-administrated group, these enzymes remained at a significantly lower level than those in the DOX group and were similar to those in both the control and Ber groups. The protective effect of CBW was further verified through histopathological analysis. DOX-exposed cardiac tissue showed collagen accumulation in Masson’s trichrome staining. Notably, it was found to be significantly lower in mice administered CBW at a level similar to that of the Ber group (Figure 5D). Likewise, the number of TUNEL-positive cardiomyocytes decreased in the CBW group compared to that in the DOX-treated mice (Figure 5E). Taken together, these data indicated that CBW protected cardiomyocytes against DICT.

### 2.6. CBW Improves Cardiac Functions against DOX-Induced Cardiac Toxicity

**Figure 6:**
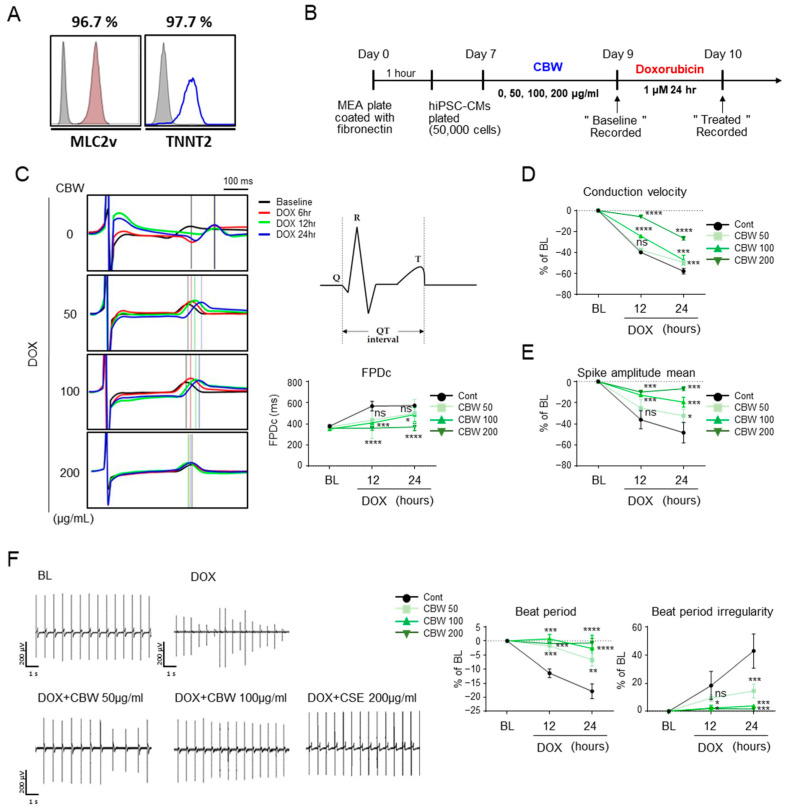
CBW reverses DOX-induced abnormal physiological changes in human-induced pluripotent stem-cell-derived cardiomyocytes (hiPSC-CMs). (**A**) Normal cord blood-cell-derived iPSCs were differentiated into cardiomyocytes. To validate hiPSC-CMs, cells were stained with the indicated antibodies, MLC2v or TNNT2, and analyzed using FACS. (**B**) Scheme of studies of hiPSC-CMs. (**C**) CBW improved the field potential in response to DOX. Field potential traces were recorded with the multielectrode array (MEA) exhibiting a mean spontaneous beating trace (**right** panel). Representative trace images were analyzed using field potential trace data (**left** panel). The values are presented as mean ± SD. ns, not significant (*p* < 0.05), * *p* < 0.05, *** *p* < 0.001, and **** *p* < 0.0001 (DOX vs. CBW) (one-way ANOVA). (**D**,**E**) CBW improved the decreased conduction velocity (**D**) and spike amplitude (**E**) by DOX treatment. Conduction velocity with percentage changes compared to that of the basal line was measured using MEA. The values are presented as mean ± SD. ns, not significant (*p* < 0.05), * *p* < 0.05, *** *p* < 0.001, and **** *p* < 0.0001 (DOX vs. CBW) (one-way ANOVA). (**F**) CBW recovered delayed beat period and irregular beat period following DOX treatment. Raw hiPSC-CM spiking data, demonstrating detection of action potential spikes at 200 μV/s. The change in the beat period and beat period irregularity with percentage changes compared to that in the baseline were measured using an MEA assay (**right** panel). The values are presented as mean ± SD. ns, not significant (*p* < 0.05), * *p* < 0.05, ** *p* < 0.01, *** *p* < 0.001, and **** *p* < 0.0001 (DOX vs. CBW) (one-way ANOVA).

Differentiated hiPSC-CMs were stained with antibodies against ventricular myosin light chain-2 (MLC2V) and troponin T2 (TNNT2) for molecular characterization (Figure 6A). The functions of hiPSC-CMs were monitored using an MEA system, and data were acquired after 9 and 10 days (Figure 6B). Field potential duration (FPD) data clearly demonstrated visible R/Q and T peaks with high signal-to-baseline ratios in cells treated with CBW before DOX exposure. As expected, DOX induced an increase in the QT interval in a time-dependent manner; however, CBW treatment prevented this increase in a dose-dependent manner (Figure 6C). Following DOX treatment, hiPSC-CMs showed a slow conduction velocity and low spike amplitude, which were recovered by CBW in a dose-dependent manner (Figure 6D and Figure 6E, respectively). Beat period and beat period irregularity values also improved in CBW-treated hiPSC-CMs (Figure 6F). Taken together, our data indicated that CBW prevented cardiac dysfunction triggered by DICT in hiPSC-CMs.

## 3. Discussion

DOX is recognized as a major contributor to the pathogenesis of heart failure, a prevalent syndrome that leads to substantial morbidity and mortality rates worldwide [26]. The five-year mortality rate of DOX-induced heart failure is estimated to be approximately 50%, indicating a worse prognosis than idiopathic or ischemic cardiomyopathy [27]. In patients with cancer undergoing anthracycline chemotherapy, dexazoxane (DEX) is the sole cardioprotective agent. However, studies on childhood lymphoma and leukemia following the use of DEX have raised concerns about its potential to diminish the anticancer activity of anthracyclines in patients with breast cancer and increase the risk of secondary malignancies [28]. Therefore, in 2017, the European Medicines Agency concluded that DEX should not be contraindicated in children at the highest risk of cardiotoxicity [29]. Therefore, the primary objective of our study was to identify materials that can prevent DICT, focusing on relatively safe food materials, and uncover the molecular mechanisms underlying their efficacy. In this study, we demonstrated, for the first time, the potential efficacy of CBW in protecting cardiomyocytes against DOX-induced toxicity, offering a novel alternative to counteract its adverse effects.

We observed that CBW exhibited a dose-dependent protective effect against a reduction in cell viability. This phenomenon is also reflected in morphological changes of cells and alterations in cellular ATP production. To date, numerous studies have focused on the anticancer effects of CBW; however, its potential to protect cardiomyocytes against DICT has not been reported. Interestingly, when the same conditions were applied to perform WST-1 assay using MDA-MB-231 cells, no inhibition of the reduction was observed in response to CBW, unlike that in H9c2 cells. This suggests that CBW does not affect the anticancer property of DOX.

Structurally, DOX contains quinone groups, and semiquinone produced by the one-electron reduction of its quinone moiety can easily react with oxygen radicals, inducing excessive oxidative stress [8]. ROS generated by DOX primarily targets the mitochondria. Compared to the number of mitochondria targeted by DOX in other tissues, that in cardiomyocytes increases by 35–40%, which makes cardiomyocytes susceptible to DOX-induced damage. Consistent with previous findings, our data clearly demonstrated an increase in intracellular ROS, a decrease in mitochondrial membrane potential, and a consequent decline in OCR following DOX treatment. DOX induces the opening of mitochondrial permeability transition pores (mPTPs), leading to induction of mitochondrial permeability transition [30]. This phenomenon results in the loss of electrochemical gradients across the inner mitochondrial membrane, and in the human myocardium, DOX-induced mPTPs opening inhibits mitochondrial respiration [31]. Our results showed that CBW alleviated DOX-induced exacerbation of mitochondrial dynamics. As previously emphasized, ROS are representative regulators of mitochondrial dynamics [32].

Intracellular ROS are controlled by antioxidant enzymes. In a state of normal physiological function, a delicate equilibrium exists between ROS generation and capacity of the antioxidant system [33]. However, if antioxidant detoxification systems become unable to effectively regulate and maintain ROS levels within tolerance limits, various cellular damages occur, ultimately resulting in cell death [34]. Continuous exposure to high concentrations of DOX triggers this phenomenon. Our findings are consistent with those of previous reports [22,25], which have demonstrated that DOX administration leads to a notable decrease in the expression of *Sod1*, *Sod2*, and *HO-1*. *Capsella bursa-pastoris* (L.) seeds increase 2,2-diphenyl-1-picrylhydrazyl radical-scavenging activity and ferric ion-reducing antioxidant potential [35]. These results strongly support the fact that *Capsella bursa-pastoris* (L.) has antioxidant potential. According to our observation, CBW induced the upregulation of SOD, especially SOD2, and HO-1 through the Nrf2 signaling pathway. Contrary to our expectations based on mRNA expression results, the expression of SOD1 showed an increase only in the 200 µg/mL CBW treatment group compared to the DOX-alone treatment group. Unfortunately, at our current level, we cannot scientifically explain these inconsistencies perfectly; however, we believe that they may be attributed to the characteristics of natural products, which often have a wider range of targets compared to single compounds. Furthermore, there is also a possibility that CBW may regulate the expression of SOD in a more specific manner than SOD1. Nrf2 plays a pivotal role in regulating several antioxidant enzymes, including SOD1 and SOD2 [36,37], by mitigating oxidative stress and facilitating the detoxification process [38]. Experimental evidence has indicated p38 MAPK-dependent Nrf2 stabilization [39,40]. All SOD isoforms possess common binding sites for various transcription factors including nuclear factor kappa beta, specificity protein 1 (Sp-1), CCAAT-enhancer-binding protein, and activator proteins 1 and 2 [41]. These transcription factors exert comparable regulatory effects on all SOD genes. Under stress conditions, p38 induces Sp-1 activation through its phosphorylation, resulting in enhanced expression of genes regulated by Sp-1 [42]. However, it appears that CBW has a direct impact on the stabilization of Nrf2 without involving p38.

We hypothesized that the most important question was whether the inhibitory mechanism of CBW against DICT in vitro translates into a cardioprotective effect in vivo. We introduced a DICT mouse model to investigate the cardioprotective effects of CBW [43,44]. Furthermore, based on MEA, we observed changes in electrophysiological indicators that affected cardiac function in hiPSC-CMs ex vivo. CBW administration resulted in low levels of toxicity indicators CK and LDH in the blood despite DOX injection. Additionally, it suppresses sclerosis and apoptosis in mouse myocardial cells. As a result, it normalized the abnormal ECG profiles caused by the DICT. The inhibitory effects of food-derived single compounds on DICT have been reported. Berberine, which was used as a positive control in this study, ameliorates DICT via the SIRT1-p66Shr pathway [45]. Fisetin demonstrates cardioprotective potential against DICT by inhibiting various signaling pathways, including oxidative stress, inflammation, and apoptosis [46]. β-LAPachone attenuates DICT by regulating autophagy via the AMPK/Nrf2 signaling pathway [47]. While previous studies have partially demonstrated the cardioprotective effects of various substances in in vivo models of DICT, they suffered from limitations. Mouse and human hearts are different, starting from their development to their responsiveness to drugs [48,49,50]. For example, notable differences in terms of cell size, duration of action potential, and beat rate are noticed. Our study overcomes the limitations of previous studies by utilizing iPSC-CMs that closely resemble human cardiac cells and provide a representative model of the human heart. Similar to the in vivo mouse model, CBW ameliorated the effects of DICT in hiPSC-CMs. Therefore, CBW intake may show cardioprotective effects in humans.

**Figure 7:**
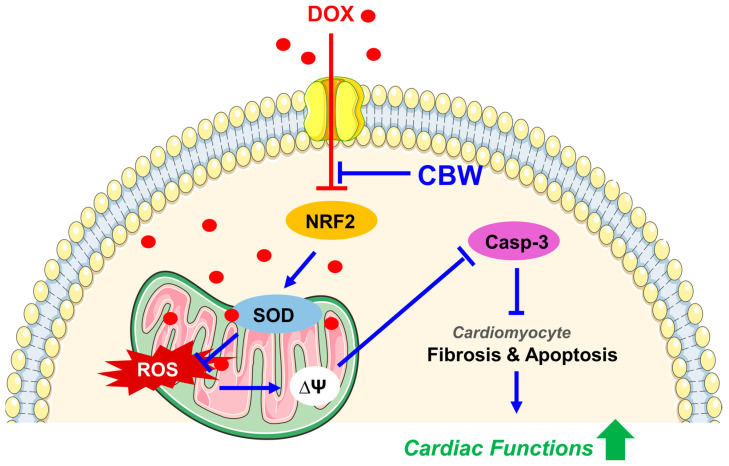
CBW ameliorates DOX-induced cardiotoxicity (DICT) by inhibiting the DOX-induced Nrf2 reduction which consequently increases the mRNA and protein expression of antioxidant enzymes, SOD1 and SOD2. This increased SOD activity suppresses ROS generation in mitochondria; hence, the DOX-induced disruption of mitochondrial membrane potential and respiration is subsequently restored. Furthermore, CBW administration inhibits collagen accumulation in cardiomyocytes and blocks apoptotic cell death, leading to protecting cardiac functions against DICT. Abbreviations: DOX, doxorubicin; CBW, water extract of *Capsella bursa-pastoris*; NRF2, nuclear factor erythroid-2-related factor 2; SOD, superoxide dismutase; ROS, reactive oxygen species; Casp-3, Caspase-3.

In this study, we elucidated for the first time that CBW ameliorates DICT via antioxidative effect mediated by Nrf2 activation, implying its potential as a natural cardioprotective therapeutic agent (Figure 7). However, this study has some limitations. First, we could not define the specific compounds of CBW which are responsible for inhibiting DICT. Second, we were unable to delve into the detailed molecular mechanisms underlying this phenomenon. Lastly, towing to various challenges associated with utilizing in vivo and ex vivo models, we were unable to adequately perform mechanistic validations based on these models. However, CBW holds immense potential to be used as a cardioprotective agent against DICT.

## 4. Materials and Methods

### 4.1. Plant Materials, Preparation, and Extraction

*Capsella bursa-pastoris* was purchased from an agricultural product brand in the Republic of Korea. The washed raw materials (2 kg) were extracted with hot water for 2 h (vacuum enriched extraction, 95 °C) and centrifuged at 8000× *g* for 30 min. The extract was then freeze-dried using a vacuum-tray freeze dryer and stored at 20 °C until use.

### 4.2. Ultra-Performance Liquid Chromatography (UPLC) Triple Quadrupole Mass Spectrometry (TQ/MS) (UPLC-TQ/MS) Analysis

UPLC-TQ/MS analyses were performed using an Agilent 1290 Infinity (Agilent Technologies, Palo Alto, CA, USA) coupled with a SCIEX 4500 TQ/MS (Sciex, Framingham, MA, USA) equipped with an electrospray ionization (ESI) source. Chromatographic separation was performed using an Acquity UPLC HSS T3 column (2.1 mm × 5 mm, 1.7 μm; Waters, Milford, MA, USA). The column was maintained at 40 °C, and a binary gradient separation was performed at a flow rate of 0.45 mL/min. Mobile phase A consisted of 0.1% formic acid in water, and mobile phase B consisted of 0.1% formic acid in acetonitrile. The gradient profile was a 0–1 min linear increase in B from 1 to 5%, a 1–3 min linear increase in B from 5 to 25% a 3–4.8 min linear increase in B from 25 to 35%, a 4.8–5.8 min hold at 35% B, a 5.8–6.8 min linear increase in B from 35 to 45% a 6.8–7.8 min hold at 45% B a 7.8–8.8 min linear increase in B from 45 to 60% an 8.8–9.3 min linear increase in B from 60 to 100% a 9.3–10 min linear increase in B from 1–100% and a 10–13 min (post-acquisition time) starting mobile phase 1% B to re-equilibrate the column. The temperature values of the autosampler and column oven were 4 and 40 °C, respectively.

MRM conditions were optimized by infusing each standard (100 ng/mL in 50% methanol) into the ESI source. The retention times and MRM transitions of the compounds are summarized in Table S1. Working standard solutions at the desired concentration for generating a calibration curve were prepared by serial dilution with an ethanol/water solution (70:30, *v*/*v*) when necessary. All stock solutions were stored at −20 °C. Calibration curves were generated using linear least squares regression analysis based on the ratio of peak areas of the analyte to that of the internal standard (IS). The equations and linear regression coefficients of the calibration curves are summarized in Table S2.

### 4.3. Cell Culture and hiPSC-CM Differentiation

H9c2 (rat myoblasts) and MDA-MB-231 (human breast carcinoma cells) were purchased from American Type Culture Collection (Manassas, VA, USA). H9c2 cells were maintained in Dulbecco’s Modified Eagle Medium (DMEM; Gibco-BRL, Gaithersburg MD, USA), and MDA-MB-231 cells were cultured in Roswell Park Memorial Institute (RPMI) 1640 medium (Gibco-BRL) supplemented with 10% fetal bovine serum (FBS; Gibco-BRL) and 1% antibiotic–antimycotic (Gibco-BRL) in a humidified chamber with 5% CO_2_ at 37 °C.

The normal iPSC line CMC_hiPSC_011 was deposited at the National Stem Cell Bank of Korea (Korea National Institute of Health) and was originally provided by the Catholic University (Seoul, Republic of Korea). To induce mesoderm differentiation of human iPSCs, cells were cultured in differentiation medium [RPMI 1640 medium (A1895601; Gibco-BRL) supplemented with 2% B-27 supplement without insulin (12587-010; Thermo Fisher Scientific, Waltham, MA, USA)] containing 8 μM CHIR99021, a small molecule activating canonical the Wnt/β-catenin pathway (4423; Tocris, Bristol, UK). After three days, the medium was replaced with a new differentiation medium that contained 2 μM C59 (Wnt antagonist) (S7037; Selleckchem, Huston, TX, USA). After two days, cells were cultured in cardiomyocyte (CM) medium (RPMI 1640 medium supplemented with 2% B-27 supplement [17504044; Gibco-BRL]) to induce cardiac maturation of progenitor cells.

### 4.4. Cell Viability

To evaluate the cytotoxicity of CBW, H9c2 or MDA-MB-231 cells were seeded in 96-well plates at 1 × 10^4^ cells/well and incubated for 24 h. Then, cells were treated with CBW for 24 h. To investigate the cytoprotective effect of CBW against DOX treatment, cells were exposed to CBW for 24 h, followed by treatment with 2 μM DOX for 48 h. Cells were reacted with 10 µL of a water-soluble tetrazolium salt (WST-1) solution (Enzo Life Sciences, Inc., Farmingdale, NY, USA) for 2 h. Then, absorbance was measured at 450–650 nm using a microplate reader (Molecular Devices, Sunnyvale, CA, USA).

### 4.5. ROS Detection

The medium was replaced with FluoroBrite DMEM containing 5% FBS and 5 µM MitoSOX Red Mitochondrial Superoxide Indicator (Invitrogen, Waltham, MA, USA). After incubation for 30 min at 37 °C, cells were washed with phosphate-buffered saline (PBS) and incubated with 200 μM MitoTracker Green probe (Invitrogen) for 30 min at room temperature (RT). Mitochondrial ROS generation was visualized using a Nikon ECLIPSE 80i microscope (Nikon, Tokyo, Japan) equipped with DS-Qi1Mc (Nikon).

### 4.6. Immunoblot Analysis

Cell extracts were prepared using M-PER Lysis buffer (Thermo Fisher Scientific) containing phosphatase and protease inhibitors (Roche, Basel, Switzerland) and incubated for 5 min at RT. The lysates were centrifuged at 20,000× *g* for 20 min at 4 °C. Total lysates were used for immunoblotting. The antibodies used in this study are listed in Supplementary Table S3. Protein bands were visualized using an imaging system (Vilber Lourmat, ZAC de Lamirault, France). Protein expression was normalized with respect to that of β-actin.

### 4.7. Cellular ATP Levels

Cellular ATP levels were determined using an ATP assay kit (Abcam, Cambridge, UK), according to the manufacturer’s instructions. In brief, H9c2 cells were seeded in a 6-well plate at a density of 2 × 10^5^ cells/well, treated 50, 100, or 200 μg/mL CBW for 24 h, and then incubated with 2 μM DOX for 48 h at 37 °C. Harvested cells were lysed with ATP assay buffer, and lysates were incubated with ATP reaction mixture for 30 min at RT in the dark. Cellular ATP levels were measured at 570 nm.

### 4.8. Quantitative Real-Time Polymerase Chain Reaction (PCR)

Total RNA was extracted from cells using RNAiso Plus (Takara, Kusatsu, Shiga, Japan). Reverse transcription was performed using a cDNA synthesis kit (Toyobo, Osaka, Japan) according to the manufacturer’s instructions. Genes were amplified using a CFX Connect Real-Time PCR detection system (Bio-Rad, Hercules, CA, USA) with SYBR Green PCR Master Mix (Roche, Mannheim, Germany). The primers used in this study are listed in Table S4, and bands were quantified with respect to Gapdh expression.

### 4.9. Mitochondrial Respiration

The mitochondrial oxygen consumption rate (OCR) was measured using a Seahorse XFe96 Analyzer (Agilent, Santa Clara, CA, USA). Briefly, H9c2 cells were seeded at a density of 1 × 10^4^ cells/well in Seahorse XF cell culture microplates. After treatment, DMEM was replaced with Seahorse XF DMEM containing 10 mM glucose, 2 mM glutamine, and 1 mM pyruvate. Then, 1.5 μM oligomycin, 2 μM p-triflouromethoxyphenylhydrazone (FCCP), and 0.5 μM rotenone/antimycin A were sequentially injected into the port of a hydrated cartridge to determine basal respiration, ATP production, and maximal respiration during the assay. The OCR was measured in real time.

### 4.10. Determination of Mitochondrial Membrane Potential

To investigate mitochondrial depolarization, H9c2 cells were treated with 100 nM tetramethylrhodamine, methyl ester, perchlorate (TMRM; Invitrogen) solution and 1 μg/mL Hoechst 33342 (Invitrogen) for 30 min at 37 °C. Mitochondrial membrane potential was measured using a Nikon ECLIPSE 80i microscope (Nikon) equipped with a DS-Qi1Mc (Nikon).

### 4.11. Fluorescence-Activated Cell Sorting (FACS) Analysis

Apoptotic cells were quantitatively analyzed by flow cytometry using Annexin V-fluorescein isothiocyanate (FITC) (640906, BioLegend, Sandiego, CA, USA), following the manufacturer’s protocol. Briefly, H9c2 cells were cultured in a 6-well plate at a density of 1 × 10^6^/well. Cells were treated with CBW (0, 50, 100, or 200 μg/mL) and 2 μM doxorubicin for 24 h. Then, cells were washed with PBS and stained with Annexin V-FITC and Zombie NIRTM fixable dye (423105, BioLegend) for 15 min at RT (25 °C) in the dark. Apoptosis was measured by quantifying the population of Annexin V-FITC-positive cells. The data of flow cytometry were plotted and analyzed using an LSR II flow cytometer (BD Biosciences, San Jose, CA, USA) and FlowJo v.10.0.7 software (Tree Star. Inc., Ashland, OR, USA).

### 4.12. In Vivo Cardiotoxicity Model

As per the International Animal Care and Use Committee guidelines, the protocols for the care of animals were approved by the Korea Food Research Institute (IACUC Approval No. KFRI-M-22004). Eight-week-old C57BL/6 mice were acclimated for one week before the experiment and randomly divided into four groups (*n* = 3 or 4/group). They were maintained in a room at 40–60% humidity, 23 ± 2 °C temperature, and 12 h light/dark cycle. To induce cardiotoxicity, 5 mg/kg DOX was injected intraperitoneally (i.p.) for four weeks (cumulative dose, 20 mg/kg). They were orally administered CBW (400 mg/kg) and berberine (40 mg/kg) five days a week. Berberine was used as a positive control. Electrocardiograms (ECG) were analyzed using EzCG Analysis Software (Ver. 7.0; BIOPAC Systems Inc., Goleta, CA, USA) two days prior to sacrifice. Blood and cardiac tissues were obtained from mice to assess DOX-induced cardiac injury.

### 4.13. Blood Test

Blood was centrifuged at 3000× *g* for 10 min at 4 °C for serum separation. The serum levels of creatine kinase (CK), lactate dehydrogenase (LDH), aspartate aminotransferase (AST), and alanine aminotransferase (ALT) were determined using DRI-CHEM 3500s (FUJIFILM, Tokyo, Japan), in accordance with the manufacturer’s protocol.

### 4.14. Masson’s Trichrome Staining

Mouse cardiac specimens were fixed in 4% buffered formalin, embedded in paraffin, and cut into 4–5 μm thick sections. Masson’s trichrome staining was performed using an MT kit (StatLab, American MasterTech, Lodi, CA, USA) in accordance with the manufacturer’s instructions. After deparaffinization and rehydration, the sections were immersed in Bouin’s fluid at 4 °C for 1 h, stained with Weigert’s hematoxylin, incubated in Biebrich scarlet acid fuchsin phosphomolybdic/phosphotungstic acid, aniline blue stain, and then fixed with 1% acetic acid. The stained slides were scanned using a PANNORAMIC Digital Slide Scanner (Gaia Science, E Pasir, Singapore), and images were captured using Slide Converter (3DHISTECH Ltd., Budapest, Hungary).

### 4.15. Terminal Deoxynucleotidyl Transferase dUTP Nick-End Labeling (TUNEL) Assay

To quantify cardiomyocyte apoptosis, TUNEL assay was performed using an in situ Cell Death Detection Kit (Promega, Madison, WI, USA). Briefly, the sections were incubated with 20 µg/mL proteinase K for 15 min at RT and then labeled with terminal deoxynucleotidyl transferase and a nucleotide mixture in reaction buffer (label solution mixture) at 37 °C for 1 h, blocked with stop/wash buffer, and incubated with peroxidase-conjugated anti-digoxigenin antibody for 30 min at RT. DNA fragmentation was visualized using diaminobenzidine (Sigma-Aldrich, St. Louis, MO, USA). Hematoxylin was used for nuclear counterstaining. The stained slides were scanned using a PANNORAMIC Digital Slide Scanner (3DHISTECH Ltd., Budapest, Hungary), and images were captured using a Slide Converter (3DHISTECH Ltd.).

### 4.16. MEA

We used an MEA data acquisition system (Maestro Edge; Axion BioSystems, Atlanta, GA, USA) for the analysis. MEA plates contained a matrix of titanium nitride electrodes with an interelectrode. MEA plates were sterilized with 70% ethanol and coated with Matrigel (354277; Corning, Corning, NY, USA). Beating cardiomyocytes were plated in CM medium in the middle of an MEA plate for at least three days. On the day of the experiment, recordings were performed for 5 min at baseline and 10 min after applying DOX. The field potential signals were analyzed for beat period means, beat irregularities, field potential duration (FPD), and spike amplitudes. FPD was normalized to the beat rate using the Fridericia formula [corrected FPD (FPDc)]. Data were analyzed using the Cardiac Analysis Tool v.3.2.2 (Axion BioSystems, Atlanta, GA, USA).

### 4.17. Statistical Analysis

All data are expressed as mean ± standard deviation (SD). One-way analysis of variance (ANOVA) was used to determine statistical significance, followed by Dunnett’s post-hoc test. Statistical significance was defined as * *p* < 0.05, ** *p* < 0.01, *** *p* < 0.001, and **** *p* < 0.0001.

## Figures and Tables

**Table 1:** Concentration of metabolites measured in CBW by UPLC-TQ MS/MS.

Compounds	Concentration (µg/g)
Apigenin-6,8-di-C-glucoside (vicenin-2)	30.92
Isoorientin	4.26
Quercetin 3-O-glucoside (isoquercitrin)	131.22
Luteolin-7-O-glucoside	133.41
Isorhamnetin-3-rutinoside (Narcissoside)	5.81
Quercetin 3-O-(6″-acetyl-glucoside)	4.37
Chrysoeriol-7-O-glucoside (Thermopsoside)	18.93

## Data Availability

Data sharing not applicable.

## Supplementary Materials

The following supporting information can be downloaded at: https://www.mdpi.com/article/10.3390/ijms242115912/s1.
