# Supplementary material for: Water Extract of Capsella bursa-pastoris Mitigates Doxorubicin-Induced Cardiotoxicity by Upregulating Antioxidant Enzymes
Source: Int J Mol Sci. 2023 Nov 2;24(21):15912. doi: 10.3390/ijms242115912 (PMC10648471; doi:10.3390/ijms242115912)
Supplement: Supplementary file 1 [file ijms-24-15912-s001.zip › ijms-2656598-supplementary.pdf]

Water extract of *Capsella bursa-pastoris* mitigates doxorubicin-induced cardiotoxicity by upregulating antioxidant enzymes

Supplementary information: Table S1 – S4

Table S1. Selected ion groups of metabolites quantified from CBW by UPLC-QTOF MS.

Compound	Retention time (min)	Ionization Mode	Precursor ion (m/z)	Quant. ion (*m/z*)	Confirm. ion (*m/z*)
Apigenin-6,8-di-C-glucoside (vicenin-2)	4.0	+	595	457	325
Isoorientin	4.3	-	447	357	327
Quercetin 3-o-glucoside (isoquercitrin)	4.8	+	465	303	-
Luteolin-7-O-glucoside	4.9	+	449	287	153
Isorhamnetin-3-rutinoside (Narcissoside)	5.1	+	625	317	479
Quercetin 3-O-(6"-acetyl-glucoside)	5.3	+	507	303	-
Chrysoeriol-7-O-glucoside (Thermopsoside)	5.5	+	463	301	286
$^{13}C_3$-Catechin (IS)	3.8	+	294	140	124
		-	292	124	109

*IS: Internal standard

Table S2. UPLC-QTOF MS.calibration curve equations and linear correlation coefficients (R^2) for metabolites quantified in CBW samples.

**Compounds**	**Con. Range (μg/g)**	**Calibration equations**	R^2
Apigenin-6,8-di-C-glucoside (vicenin-2)	1-100	y = 9.2882x – 0.1018	0.9991
Isoorientin	1-100	y = 3.0411x + 0.0656	0.9953
Quercetin 3-o-glucoside (isoquercitrin)	1-500	y = 0.1152x – 0.0115	0.9934
Luteolin-7-O-glucoside	10-1000	y = 1.1906x – 0.0439	0.9967
Isorhamnetin-3-rutinoside (Narcissoside)	1-100	y = 6.2196x + 0.8441	0.9956
Quercetin 3-O-(6"-acetyl-glucoside)	0.5-50	y = 2.7691x + 0.0364	0.9925
Chrysoeriol-7-O-glucoside (Thermopsoside)	5-500	y = 2.1468x + 0.0288	0.9987

Table S3. Antibodies used in this study

Antibody	Vendor<br>Cat no.	Local	Country	Titer	Usage
**aSOD1**	cell signaling<br>37385	Danvers, MA	USA	1:4000	
**aSOD2**	cell signaling<br>13141	Danvers, MA	USA	1:5000	
**aCatalase**	cell signaling<br>14097	Danvers, MA	USA	1:3000	
**aHO-1**	cell signaling<br>43966	Danvers, MA	USA	1:3000	
**ap-JNK**	cell signaling<br>4668	Danvers, MA	USA	1:3000	
**aJNK**	cell signaling<br>9252	Danvers, MA	USA	1:3000	
**ap-p38**	cell signaling<br>4511	Danvers, MA	USA	1:3000	
**ap38**	cell signaling<br>9212	Danvers, MA	USA	1:3000	W.B
**ap-ERK**	cell signaling<br>8544	Danvers, MA	USA	1:3000	
**aERK**	cell signaling<br>4695	Danvers, MA	USA	1:3000	
**aNRF1**	cell signaling<br>46743	Danvers, MA	USA	1:3000	
**aNRF2**	cell signaling<br>20733	Danvers, MA	USA	1:3000	
**aCleaved-Caspase3**	cell signaling<br>9661	Danvers, MA	USA	1:3000	
**aPARP**	cell signaling<br>9542	Danvers, MA	USA	1:3000	
**ab-actin**	cell signaling<br>4967	Danvers, MA	USA	1:3000	

Abbreviation: αSOD1, anti-Superoxide dismutase1; αSOD2, anti-Superoxide dismutase2; αHO-1, anti-Heme oxygenase-1; αp-JNK, anti-phospho-c-Jun N-terminal kinase; αJNK, anti-c-Jun N-terminal kinase; αp-p38, anti-phospho-p38 Mitogen-activated protein kinase; αp38, anti-p38 Mitogen-activated protein kinase; αp-ERK, anti-phospho-Extracellular signal-regulated kinase; αERK, anti-Extracellular signal-regulated kinase; αNRF1, anti-Nuclear respiratory factor1; αNRF2, anti-Nuclear factor erythroid-derived-like 2; αPARP, anti-Poly (ADP-ribose) polymerase; αβ-Actin, anti-beta Actin; W.B, Western blotting

Table S4. Primers used in this study for q-PCR

**Gene**		**Sequence (5’-3’)**	**Usage**
Gapdh	Forward	AGACAGCCGCATCTTCTTGT	qPCR
	Reverse	CTTGCCGTGGGTAGAGTCAT	
Nrf2	Forward	CTACAGTCCCAGCAGGACAT	
	Reverse	TTTGGGAATGTGGGCAACCT	
Nrf1	Forward	GGCGCAGCACCTTTGGAGAATGTG	
	Reverse	CATCGATGGTGAGAGGGGGCAGTTC	
Sod1	Forward	TGTGTCCATTGAAGATCGTGTG	
	Reverse	CTTCCAGCATTTCCAGTCTTTG	
Sod2	Forward	GGACAAACCTGAGCCCTAAG	
	Reverse	CAAAAGACCCAAAGTCACGC	
Ho-1	Forward	GTCCCAGGATTTGTCCGAGG	
	Reverse	GGTACAAGGAGGCCATCACC	

Abbreviation: *Gapdh*, Glyceraldehyde-3-phosphate dehydrogenase; *Nrf2*, Nuclear factor erythroid-derived-like 2; *Nrf1*, Nuclear respiratory factor1; *Sod1*, Superoxide dismutase1; *Sod2*, Superoxide dismutase2; *Ho-1*, Heme oxygenase-1; qPCR, Quantitative polymerase chain reaction.
